# A loss of function mutation in *CLDN25* causing Pelizaeus-Merzbacher-like leukodystrophy

**DOI:** 10.1093/hmg/ddae038

**Published:** 2024-03-17

**Authors:** Yosuke Hashimoto, Claude Besmond, Nathalie Boddaert, Arnold Munnich, Matthew Campbell

**Affiliations:** Smurfit Institute of Genetics, Trinity College Dublin, D02 VF25, Dublin, Ireland; INSERM UMR1163, Institut Imagine, Paris University, F-75015, Paris, France; Clinical Genetics Department, Necker Hospital, APHP Centre-Paris University, F-75015, Paris, France; INSERM UMR1163, Institut Imagine, Paris University, F-75015, Paris, France; Department of Pediatric Radiology, Hospital Necker Enfants Malades, F-75015, Paris, France; INSERM UMR1163, Institut Imagine, Paris University, F-75015, Paris, France; Clinical Genetics Department, Necker Hospital, APHP Centre-Paris University, F-75015, Paris, France; Smurfit Institute of Genetics, Trinity College Dublin, D02 VF25, Dublin, Ireland; FutureNeuro, Science Foundation Ireland Research Centre for Chronic and Rare Neurological Diseases, Royal College of Surgeons in Ireland, University of Medicine and Health Sciences, Dublin, Ireland

**Keywords:** Claudin-25, Leukodysdrophy, tight junction, ZO-1

## Abstract

Claudin-25 (CLDN-25), also known as Claudin containing domain 1, is an uncharacterized claudin family member. It has less conserved amino acid sequences when compared to other claudins. It also has a very broad tissue expression profile and there is currently a lack of functional information from murine knockout models. Here, we report a *de novo* missense heterozygous variant in *CLDN25* (c. 745G>C, p. A249P) found in a patient diagnosed with Pelizaeus-Merzbacher-like leukodystrophy and presenting with symptoms such as delayed motor development, several episodes of tonic absent seizures and generalized dystonia. The variant protein does not localize to the cell-cell borders where it would normally be expected to be expressed. Amino acid position 249 is located 4 amino acids from the C-terminal end of the protein where most claudin family members have a conserved binding motif for the key scaffolding protein ZO-1. However, CLDN-25 does not contain this motif. Here, we show that the C-terminal end of CLDN-25 is required for its junctional localization in a ZO-1 independent manner. The A249P mutant protein as well as a deletion mutant lacking its last 5 C-terminal amino acids also failed to localize to the cell-cell border *in vitro*. Intriguingly, cellular knockout of *CLDN25*, *in vitro*, appeared to increase the integrity of the tight junction between 2 contacting cells, while driving highly unusual increased movement of solutes between cells. We propose that the barrier function of CLDN-25 is akin to a decoy claudin, whereby decreasing its expression in “leaky” epithelial cells and endothelial cells will drive dynamic changes in the adhesion and interaction capacity of cell-cell contact points. While it remains unclear how this *de novo* CLDN-25 mutant induces leukodystrophy, our findings strongly suggest that this mutation induces haploinsufficiency of CLDN-25. Elucidating the function of this uncharacterized claudin protein will lead to a better understanding of the role of claudin proteins in health and disease.

## Introduction

The hypomyelinating X-linked leukodystrophy Pelizaeus-Merzbacher disease (PMD) is characterized by nystagmus, impaired motor development, ataxia, choreoathetotic movements, dysarthria, and progressive spasticity. Commonly, patients presenting with these conditions will have pathogenic variants in the *PLP1* gene, which encodes a major myelin component, proteolipid protein 1 (PLP1). *PLP1* gene duplications mediate overexpression and missense mutations-mediate disruption of normal PLP1 processing and intracellular trafficking, causing loss of oligodendrocytes [1]. Patients with this same PMD phenotype but without pathogenic variants in *PLP1* are considered to have Pelizaeus-Merzbacher-like disease (PMLD). Major causative pathogenic variants for PMLD are variants in *GJC2* (also known as *GJA12*) (encoding connexin 47) [2] and minor ones are variants in *HSPD1* (encoding mitochondrial heat-shock protein 60) [3], *SNAP29* (encoding synaptosomal-associated protein 29) [4], *RARS* (encoding arginyl-tRNA synthetase) [5], *AIMP1* (encoding aminoacyl-tRNA synthetase-interacting multi-functional protein 1) [6]. Only *PLP1* and *GJC2* are highly and selectively expressed in oligodendrocytes [7] and the other genes are expressed ubiquitously throughout the brain. The prevalence of PMLD is not well defined due to the small numbers of cases, however it can be considered an ultra-rare condition.

The third most abundant protein in myelin is claudin-11 (CLDN-11). CLDNs are four pass transmembrane proteins with two extracellular domains and form tight junctions between two adjacent plasma membranes to limit the permeation of ions and solutes. CLDNs are divided into two distinct types: classic CLDNs (CLDN-1 to -10, -14, -15, -17, and -19) and non-classic CLDNs (CLDN-11, -12, -13, -16, -18, and -20 to -27). The amino acid sequences and predicted structure/function of classic CLDNs are highly conserved, while those of non-classic CLDNs are not. As an example, CLDN-11 is able to form a tight junction via self-oligomerization by cis-interactions (within the same plasma membrane) and trans-interactions (between two adjacent plasma membranes), but most non-classic CLDNs cannot do this [8]. Therefore, most non-classic CLDNs function as tight junction proteins via cis-interactions to the other CLDNs, but these functions are still not well characterised.

Recently, *de novo* loss of function pathogenic variants in *CLDN11* were found in patients with hypomyelinating leukodystrophies not dissimilar to PMD [9]. CLDN-11 is a predominant component of tight junction strands/dots in the intramyelinic compartment of oligodendrocytes [10] and myelin lacking CLDN-11 is more diffusely permeable to solutes and ions in mice [11]. The identified CLDN-11 mutant has an extended C-terminal cytosolic domain, where interactions between CLDNs and the scaffolding protein, ZO-1 occur. Of note, *Cldn11^−/−^* mice do not exhibit hypomyelination [10] because CLDN-11 and PLP1 can compensate for each other [12]. Therefore, disrupting CLDN-11 function/localization without the loss of its expression may cause hypomyelination. The second most highly expressed CLDN in oligodendrocytes is CLDN domain containing 1 (CLDND1), more commonly known as CLDN-25 [7, 13, 14]. The function of CLDN-25 is, however, very poorly understood due to the amino acid sequences of CLDN-25 being particularly unique for a non-classic CLDNs [13]. CLDN-25 is also expressed abundantly in many tissues and cell types, but its barrier function has been assessed only in brain endothelial cells whose tight junctions are almost exclusively composed of CLDN-5 [15, 16]. There are no reports about the phenotype of *Cldn25* knockout mice.

Here, in a patient with features suggestive of Pelizaeus-Merzbacher, we find that a *de novo* missense pathogenic variant in *CLDN25* (c. 745G>C, p. A249P) shows haploinsufficiency of CLDN-25. This patient was not found to have any other pathogenic variants that may cause PMLD or other types of hypomyelinating leukodystrophy. Here, we also show that the haploinsufficiency is mediated by its C-terminus and suggest CLDN-25 is a critical and ubiquitously expressed CLDN that has dosage sensitivity as it pertains to neural integrity in humans.

## Results

### Identification of a novel pathogenic missense pathogenic variant of CLDN-25 in a patient with pelizaeus-like leukodystrophy

The patient, a boy, was the third child of healthy unrelated parents of French ancestry. He was born after a normal pregnancy and term delivery (birth weight: 3745 g, height: 54 cm, OFC: 36 cm). His older brothers are healthy, and his first four months of life were uneventful. Multidirectional nystagmus with trunk hypotonia, gradual loss of head control and oro-facial dystonia were first noted at four months. At 16 months, he was a smiling child with good social interactions. He could grab objects and toys, turn in his bed independently, straighten his arms while lying and being able to babble. Yet, his motor development milestones were markedly delayed with major trunk hypotonia, inability to sit unaided, poor head control and bouts of generalized dystonia and opisthotonos triggered by voluntary movements. Episodes of absence seizures with brief staring spells, pallor, lip cyanosis, tongue and lid clonus first occurred at the age of 4 4/12 years. Several episodes of tonic absent seizures with slow elevation of the upper limbs occurred thereafter. Electro-encephalography (EEG) displayed a low, disorganized activity with rapid rhythms, slow delta-theta waves and left fronto-centro temporal spikes.

**Figure 1:**
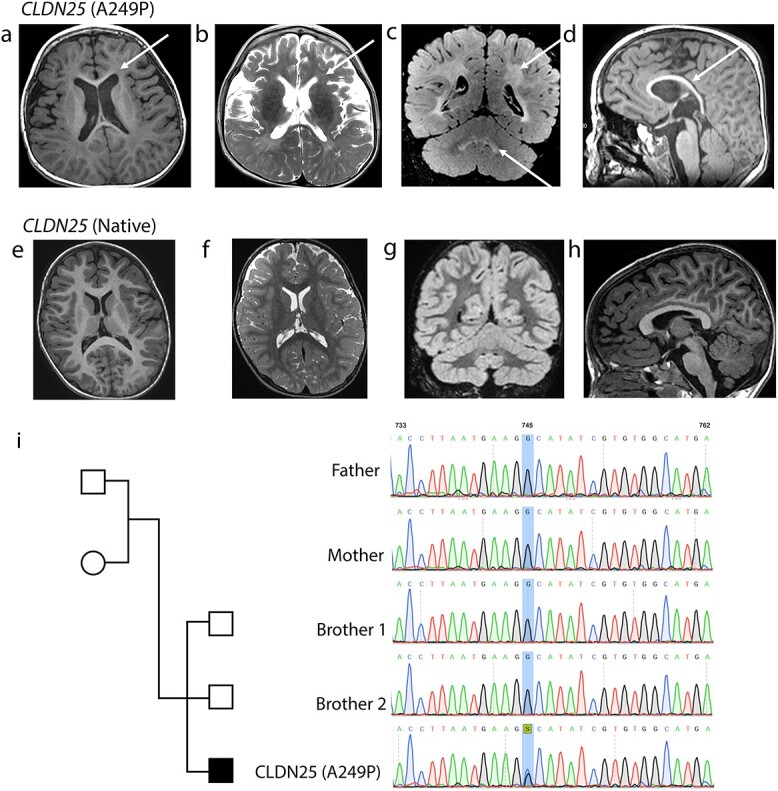
Neuroimaging of patients with leukodystrophy. (a–d) T1 and T2-weighted and FLAIR images of our patient. Neither brain atrophy or calcification is observed. Hypomyelination is represented by a low signal intensity on T1-weighted images (a and d) and a hyperintensity on T2-weighted and FLAIR images (b and c, respectively) in the periventricular and juxta ventricular white matter. FLAIR and T2 sequences also showed a hyperintensity in subcortical region and cerebellar nuclei. (e–h) T1 and T2-weighted and FLAIR images from an age matched control subject. (i) The family pedigree of the patient diagnosed with hypomyelinating leukodystrophy. Sanger sequencing chromatograms showing (c.745G>C) variant in the *CLDN25* gene.

At the age of 3 8/12 years, brain MRI showed delayed myelination for age with diffuse, supratentorial hypomyelination. T2 and Flair sequences showed subcortical and periventricular hyperintensities, mainly predominating in the parieto-temporo-occipital regions with hyperintensities of dentate nuclei, consistent with leukodystrophy (Fig. 1a–d). This was in stark contrast to an age matched control subject (Fig. 1e–h). Interestingly, brain MRI images were non-progressive with no evidence of cavitation at the age of 2 years, 3 4/12 years and 4 6/12 years.

Electro-retinography (ERG), nerve conduction velocities, visual and auditory evoked potentials, somatosensory evoked potentials, retinal fundus and CT scans were all normal. Extensive metabolic workup showed normal plasma and CSF lactate, plasma amino acid and urinary organic acid chromatography and unremarkable CGH array.

Currently, at the age of 8 6/12 years, his condition is largely stable with no evidence of significant progression. While he is not mobile, he is a smiling boy with no speech but a good understanding and an outgoing personality. He complains of limb stiffness and hip luxation. He has a major spine deformation due to progressive scoliosis. He experiences occasional unilateral clonic and absent seizures. He receives a mixed oral and enteral nutrition via gastrostomy, which is a common supportive management for patients with PMD and PMLD with severe dysphagia [17].

Whole exome sequencing identified a *de novo* heterozygous pathogenic variant in exon 5 of the *CLDN25* gene: NM_001040181.1: c.745G>C (Ala249Pro, A249P) (Fig. 1i). This variant is regarded as deleterious by the Polyphen2, Sift and Mutation Taster software.

### Functional characterization of the CLDN-25 A249P mutant

**Figure 2:**
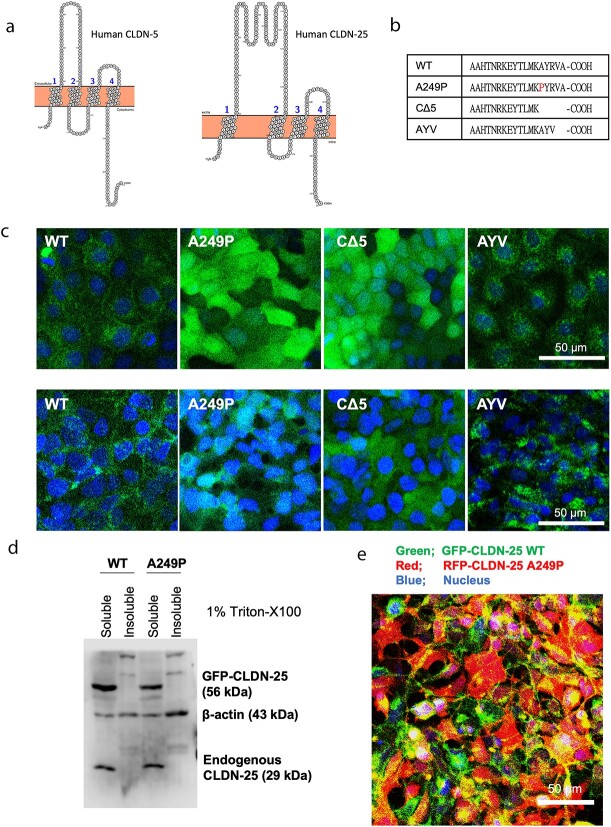
The subcellular localization of wild-type or A249P CLDN-25. (a) Structure of CLDN-25 compared to CLDN-5. The extracellular domains of CLDN-5 and -25 are shown by POTTER (http://wlab.ethz.ch/protter/start/). (b) The amino acid sequences of C-terminal domain of CLDN-25 wild-type (WT) or its mutants (A249P, CΔ5 or AYV) are shown. (c) The subcellular localization of GFP-CLDN-25 WT or its mutants in stable transfectants of MDCKII cells or d) HEK293 cells (green; GFP-CLDN-25 s, blue; nuclei. Bar, 50 μm). (e) Detergent solubility of CLDN-25. HEK293 cells expressing GFP-CLDN-25 WT or A249P mutant was extracted by 1% triton-X (soluble fraction) followed by 2% SDS (insoluble fraction). GFP-CLDN-25 and endogenous CLDN-25 was detected by western blotting. (f) HEK293 cells stably expressing GFP-CLDN-25 WT and RFP-CLDN-25 A249P were prepared. green; GFP-fused protein, red; RFP-fused protein, blue; nuclei. Bar, 50 μm.

Although CLDN-25 has a conserved CLDN signature, it has a completely different amino acid sequence and protein structure from the other CLDN family members (Fig. 2a). It has 3 additional extracellular segments in the first extracellular domain and doesn’t have a conserved cis-polymerization motif in its extracellular domains; one or two bulky hydrophobic amino acids (M, L, or I) in an extracellular helix in the first extracellular domain interact with a hydrophobic pocket composed of two aromatic amino acids (F, Y and W) in the second extracellular domain for cis-polymerization (Supplementary Fig. S1) [18, 19]. CLDN-25 also lacks conserved palmitoylation sites in its C-terminal cytoplasmic domain for efficient junctional localization [20]. Added to this, the C-terminal end of CLDN-25 is A; it does not have a hydrophobic V at the C-terminal end which it would need to interact with the PDZ1 domain of the large scaffolding protein ZO-1 [21]. Amino acid position 249 is located in C-terminal cytosolic domain and the amino acid sequences around there are highly conserved among species (Supplementary Fig. S2).

To examine whether the A249P mutation in the *CLDN25* gene is a pathogenic mutation, the subcellular localization of CLDN-25 A249P in MDCKII cells was elucidated and we also generated non-functional C-terminus constructs (Fig. 2b). Wild-type (WT) CLDN-25 is known to localize at cell-cell borders with weak expression in the cytoplasm of MDCKII cells, HEK293 cells and endothelial cells [13, 15, 16]. As it is a CLDN protein, as expected, GFP-tagged CLDN-25 WT was weakly localized at the cell-cell border (Fig. 2c); however, GFP-tagged mutant CLDN-25 A249P was not, showing that the mutant protein was not able to localize to the cell-cell border. This lack of proper cellular localization was phenocopied in a deletion mutation that lacked 5 amino acids (CΔ5) from the C-terminus. The CLDN-25 mutant with a canonical YV-COOH motif (AYV) showed a cytoplasmic, non-junctional localization pattern, indicating that a forced interaction to ZO-1 did not induce junctional stabilization of CLDN-25. The abnormal localization pattern of CLDN-25 mutants was also observed in HEK293 cells (Fig. 2d), which do not express classic CLDNs, but express CLDN-25 (Fig. 2e), indicating that endogenous CLDN-25 can be localized at the cell-cell border by its C-terminal domain.

To confirm whether CLDN-25 A249P expression impacts the junctional localization of the native CLDN-25 in a dominant negative manner, HEK293 cells expressing GFP-CLDN-25 WT with RFP-CLDN-25 A249P were prepared. (Fig. 2e). RFP-CLDN-25 A249P could not affect the subcellular localization of GFP-CLDN-25 WT, suggesting that the CLDN-25 A249P mutation induces haploinsufficiency of the junctional CLDN-25 protein (Fig. 2f).

### CLDN-25 does not interact with ZO-1

**Figure 3:**
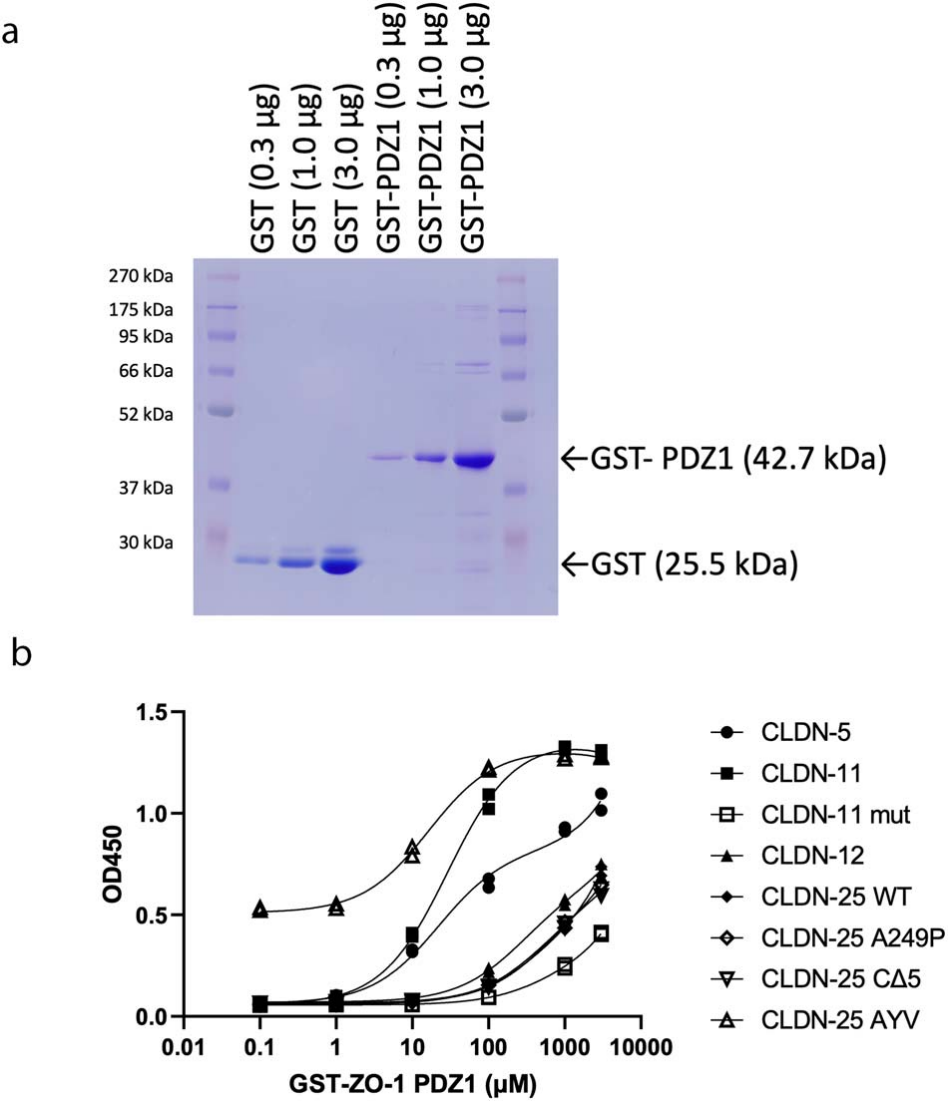
The binding affinity of C-terminal domain of CLDN-25 to ZO-1. (a) A coomassie blue stained gel image of purified recombinant GST-ZO-1 PDZ1. The apparent molecular weight of this protein is 42.7 kDa. (b) The reactivity of GST-ZO-1 PDZ1 against coated C-terminal peptides of CLDNs. GST-ZO-1 PDZ1 was titrated in duplicate. The binding affinities of these peptides to GST-ZP-1 PDZ1 in Table 1 were calculated by this titration assay.

The binding affinity of these CLDN-25 mutants against ZO-1 was estimated by using recombinant GST-ZO-1 PDZ1 and synthesized C-terminal peptides of CLDN-25 (Table 1 and Fig. 3a and b). The C-terminal peptides of CLDN-5 and -11 were used as a positive control and those of CLDN-12 and pathogenically C-terminally extended CLDN-11 [9] were used as a negative control. Unsurprisingly, CLDN-25 WT, A249P and CΔ5 could not bind to GST-ZO-1 PDZ1 and their binding affinity was equivalent to the C-terminal peptides of CLDN-12 and mutated CLDN-11. Interestingly, the synthetic CLDN-25 AYV mutant could bind to GST-ZO-1 PDZ1 with an almost equivalent affinity to CLDN-5 and -11.

**Table 1:** The binding affinity of GST-ZO-1 PDZ1 to C-terminal CLDN peptides.

	**Peptide sequences**	**K**_**D**_ **(μM) [95% CI]**
CLDN-25 WT	CWAAHTNRKEYTLMKAYRVA	386.2 [201.0–1053]
CLDN-25 A249P	CWAAHTNRKEYTLMKPYRVA	445.0 [308.4–708.3]
CLDN-25 CΔ5	CAAAAAWAAHTNRKEYTLMK	674.7 [388.7–1717]
CLDN-25 AYV	CAAWAAHTNRKEYTLMKAYV	16.47 [13.13–20.78]
CLDN-5	CYSAPRRPTATGDYDKKNYV	21.9 [12.85–40.12]
CLDN-11	CRFYYTAGSSSPTHAKSAHV	29.16 [24.08–35.42]
CLDN-11 mut	CLLVTVWGSPFLCQALKPKV	571.4 [268.1–3151]
CLDN-12	CARSRLSAIEIDIPVVSHTT	278.7 [174–492.3]

### The role of CLDN-25 in a CLDN dominant barrier

**Figure 4:**
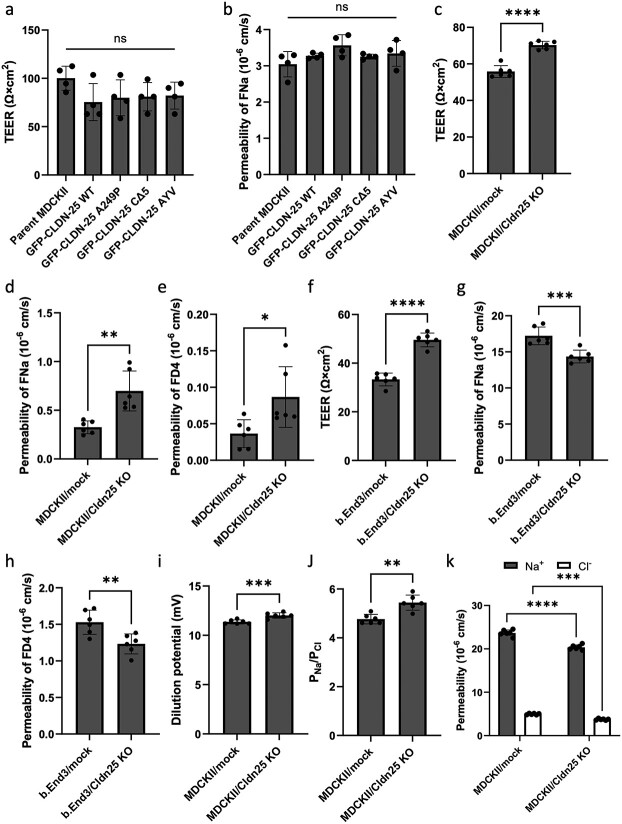
The effect of CLDN-25 expression on CLDNs-based barriers. (a and b) The barrier strength of GFP-CLDN-25 s-expressing MDCKII cells, *Cldn25* knockout MDCKII cells (c to e and i and jg) and *Cldn25* knockout b.End3 cells (f and h) was evaluated by measuring TEER (a, c and f), paracellular permeability of sodium fluorescein (FNa) (b, d and g) or 4 kDa of FITC-dextran (FD4) (e and h), dilution potential (i) permeability ratio of Na^+^ to Cl^−^ (P_Na_/P_Cl_) (j) and absolute Na^+^ or Cl^−^ permeability (k). Values represent the mean ± SD (n = 4 or 6).

Finally, to estimate how haploinsufficiency of CLDN-25 affects a CLDN dominant barrier in representative *in vitro* cell based models, trans-epithelial/endothelial electrical resistance (TEER) and solute permeability against sodium fluorescein (377 Da) and/or 4 kDa FITC-dextran (FD4) were measured. The TEER and apparent permeability of sodium fluorescein were not altered in MDCKII cells expressing GFP-CLDN-25 WT or its mutants (Fig. 4a and b). *Cldn25* knockout MDCKII cells and b.End3 cells were prepared and their TEER and solute permeability were measured (Fig. 4c to h). Intriguingly, knockout of *Cldn25* actually increased TEER in both cell types and lowered solute permeability in b.End3 cells. Inconsistently, solute permeability was increased in MDCKII/*Cldn25* KO cells, indicating that CLDN-25 is not a simple CLDN that makes tight junctions weaker. The similar barrier phenotype, increased TEER with increased paracellular flux, could be observed in MDCKII cells expressing a mutant of another barrier-forming protein, occludin (OCLN) [22]. This OCLN mutant does not have ZO-1 binding domain; it could be incorporated into the tight junctions without apparent changes in the morphology of tight junctions, but with abnormal junctional localization. To obtain more information about this complicated paracellular barrier in MDCKII/*Cldn25* KO cells, ion permeabilities were also analyzed (Fig. 4i to k). The relative cation permeability was significantly, but not largely increased in MDCKIII/*Cldn25* KO cells, indicating that CLDN-25 does not form cation- or anion-selective channel. These results suggest that CLDN-25 is not a barrier- or channel-forming CLDN and the effect of down-regulated CLDN-25 on the barrier phenotype may depend on the composition/ratio of CLDNs in the tight junctions.

## Discussion

In our patient, we see noncavitary supratentorial leukodystrophy which is in fact hypomyelination, it is stable with no atrophy, no calcification, no basal ganglia abnormalities and no lactate nor iron accumulation. The first diagnosis to evoke is *PLP1*-mutation-mediated disease (PMD) like and the plethora of diseases associated with a pattern of hypomyelination. In our patient, hypomyelination is represented by a slight hyperintensity on T2-weighted images, but also low signal intensity on T1-weighted images in the periventricular and juxta ventricular white matter. The internal capsules, corpus callosum and brainstem are normal for age, with hypointensity on T2 and marked hyperintensity on T1-weighted images. There are also hyperintensities on T2 of the cerebellar nuclei.

In classical PMD, typically, T2-weighted images show a high signal intensity of all unmyelinated white matter structures, whereas these structures have a low signal intensity on T1-weighted images [23]. In fact, no high signal intensity areas are seen on T1- weighted images. In cases of classical PMD myelin is present in the brain stem, the cerebellar white matter the posterior limb of the internal capsule, the thalamus, and the globus pallidus. The T1-weighted images show presence of myelin in the central areas. On the T2-weighted images, some myelin is seen in the cerebellum and brain stem. The pattern of myelin presence is consistent with arrest of myelination soon after birth. There may be atrophy of the cerebral hemispheres in severe PMD cases [17].

Our findings have shown that the A249P pathogenic variant in CLDN-25 impacts the cell-cell localization of the protein which is almost certainly pre-disposing to disease. The variant protein appears unable to localize to the cell-cell junctional borders and this has the potential to have numerous pathological sequalae dependent on the cell type expressing the protein. We also show that the typical cell-cell border function of CLDN-25 is likely to be very different from other CLDNs.

Classic CLDNs localize at the cell-cell border through interaction of their extracellular domains. Direct ZO-1 binding at their C-terminus is generally not necessary for their junctional localization, but ZO-1/CLDN interactions do contribute to stabilizing their junctional localization [24, 25]. As the CLDN-25 protein cannot build tight junction strands on its own, its unique extracellular domains are not able to form homophilic interactions with CLDN-25 molecules on an adjacent cell [8]. Our results have shown that only the C-terminal cytosolic domain of CLDN-25 is able to determine junctional/subcellular localization of the protein. However, neither the C-terminal end of CLDN-25 WT nor the A249P mutant could function as a ZO-1 binding domain, suggesting that this typical mode of junctional stabilization is not used by CLDN-25.

It is possible that CLDN-25 may change the morphology/complexity of tight junction strands like OCLN, which cannot form tight junctions by itself but can increase the complexity of junctional strands via interactions with CLDNs [26]. Another possibility related to CLDN-25 function is that it may affect the ability of other CLDNs to interact with the cytoskeleton by recruiting as yet unidentified proteins to ZO-1 scaffolds at the tight junction. A similar enigma was observed in *Cldn12* knockout mice, which have a compromised peripheral myelin barrier [27]. CLDN-12 also localizes at the cell–cell border [28], but it does not form homophilic trans-interactions [8] nor does it bind to ZO-1; however, its deficiency in mice changed the paracellular tightness and morphology of tight junction strands in the sciatic nerve without impacting other CLDNs [27]. Without a ZO-1 binding motif, CLDN-12 or -25 may show faster mobility at the junction and enhance the mobility of tight junction strands [28] although it remains unclear how these CLDNs are incorporated into tight junctions.

There are two major limitations in this study. Firstly, CLDN-25 is expressed ubiquitously including in non-epithelial or endothelial cells with low to intermediate expression levels [7, 14] and it is highly possible that it has important but, as yet, un-identified non-junctional functions. In the mouse brain, CLDN-25 is more highly expressed in oligodendrocytes compared to any other brain cell [7]. Therefore, its expression levels might affect the barrier/function of myelin.

While we can conclude that the CLDN-25 A249P pathogenic variant induces haploinsufficiency of CLDN-25, we have only identified one patient to date and it remains to be seen if there are more. Interestingly, our patient develops epilepsy which is an infrequent complication of PMD [17, 29]. However, it is a very common symptom for patients with pathogenic variants in barrier-forming tight junction proteins, CLDN-5 and OCLN, expressed in brain endothelial cells [30, 31] but these patients also display brain calcification in the basal ganglia and brain stem which was not observed in our patient.

Added to this, the *in vivo* function of CLDN-25 remains unknown as *Cldn25^−/−^* mice have yet to be characterized or indeed reported. Future work using oligodendrocyte precursor cells from genetically engineered mice or induced pluripotent stem cell-derived oligodendrocytes are required to study the putative role of CLDN-25 in myelination and white matter diseases. While this study represents the first description of a pathogenic mutation in *CLDN25*, it also represents the first attempt at characterizing the function of this enigmatic claudin.

## Materials and methods

### Neuroimaging

Diagnostic MRI was performed with axial and sagittal T1 weighted sequences, axial T2 weighted sequence and coronal FLAIR sequences.

### Exome sequencing

For exome sequencing, all participants provided written consent. Libraries were prepared from 3 μg genomic DNA extracted from whole blood using an optimized SureSelect Human Exome kit (Agilent). Captured, purified and clonally amplified libraries targeting the exome were sequenced on a HiSeq 2000 (Illumina). Sequence reads were aligned to the human genome (hg19) using BWA software. Downstream processing was carried out with the Genome analysis toolkit (GATK), SAMtools and Picard Tools (http://picard.sourceforge.net/). Single-nucleotide 2 variants and indels were subsequently called by the SAMtools suite (mpileup, bcftools, vcfutil). All calls with a read coverage ≤ 5× and a Phred-scaled SNP quality of ≤ 20 were filtered out. Substitution and variation calls were made with the SAMtools pipeline (mpileup). Variants were annotated with an in-house bioinformatics platform pipeline based on the Ensembl database (release 67). Mutation into *CLDN25* was confirmed by direct sequencing using BigDyedideoxy terminator chemistry and an ABI3130xl genetic DNA analyzer (Applied Biosystems) after polymerase chain reaction (PCR) using genomic DNA from a patient and his family members. Primer sequences and positions, PCR conditions and product sizes are available upon request.

### Plasmid vectors

Human gene sequence encoding CLDN-25 isoform a (NP_001035271.1) (Supplementary Fig. S2b) was synthesized by GeneArt (Thermo Fisher Science) and was inserted into pcDNA3.1 vectors (neomycin or hygromycin resistance gene coding vector). A series of CLDN-25 mutants were prepared using different reverse primers. GFP tag or RFP tag was inserted into N-terminal side of CLDN-25. pET151/D-TOPO coding 6xHis-GST-ZO-1 PDZ1 (NP_003248.3; ZO-1 isoform a, 12–118 amino acids) was synthesized by GeneArt. gRNA sequences were inserted into pU6 vectors containing puromycin resistance gene. The target sequences of gRNAs are 5′- GTAGATCAATCCCACTATTG-3′ for dog *Cldn25* and 5′-TGGCGGCCGCTCTCTTCATC-3′ for mouse *Cldn25*. All primers are listed in Supplementary Table S1.

### Stably transfected cells

Human embryonic kidney (HEK293) cells, Madin-Darby canine kidney strain II (MDCKII) and mouse immortalized brain endothelial cell line (b.End3) were maintained in Dulbecco’s modified Eagle’s medium (DMEM) supplemented with 10% fetal bovine serum (FBS) (Sigma-Aldrich), 100 U/ml penicillin, and 100 μg/ml streptomycin (Sigma-Aldrich). All cells were incubated at 37°C under 5% CO_2_.

To prepare stable transfectants expressing CLDN-25 proteins, the vectors were transfected into cells using FuGENE HP DNA (Promega) and cells were cultured for more than 14 days with 500 μg/ml of G-418 or 200 μg/ml (Promega) of hygromycin B (Sigma-Aldrich). To prepare knockout cell lines, CRISPR/Cas9 was transduced in MDCKII and b.End3 cells using LentiArray Cas9 Lentivirus (Thermo Fisher Scientific) and cells were cloned by a limiting dilution with 10 μg/ml of blasticidin S (invivogen) (MDCKII/mock and b.End3/mock cells). Then, gRNA-expressing vectors were transfected using FuGENE HP DNA and cells were cultured for more than 7 days with 5 μg/ml of puromycin (invivogen). Cells stably expressing gene of interest or lacking gene of interest were cloned by a limiting dilution method.

### Confocal microscopy

Stably transfected cells were seeded into collagen type I coated (BD biosciences) 8 chamber chamber slide (Sarstedt) and cultured for 2 days to reach the confluent. Cells were fixed by 4% paraformaldehyde for 15 minutes and were washed three times by PBS. Then, cells were counterstained with Hoechst33342 (Sigma-Aldrich) to visualize nuclei. Images were acquired on a Zeiss LSM 710 confocal microscope (Carl Zeiss).

### Preparation of ZO-1 PDZ1

*Escherichia coli* BL-21 (DE 3) was transformed with the pET151/D-TOPO vector encoding 6xHis-GST-ZO-1 PDZ1 (42.7 kDa), and protein expression was induced by the addition of isopropyl-d-thiogalactopyranoside. The cells were then harvested and lysed in buffer A [20 mM sodium phosphate (pH 7.4), 300 mM NaCl, 10 mM imidazole, 0.1 mM phenylmethanesulfonyl fluoride] by sonication. The recombinant protein was isolated from cells lysate by an immobilized metal affinity chromatography column (HisPure Ni-NTA Spin Column, Thermo Fisher Scientific). The buffer was exchanged with PBS by dialysis cassettes (Slide-A-Lyzer, 10 kMWCO, Thermo Fisher Science) and stored at −80°C. The concentration of 6xHis-GST-ZO-1 PDZ1 was quantified with a BCA protein assay kit (Thermo Fisher Scientific). The purity was checked by SDS-PAGE followed by Coomassie blue staining.

### Elisa

Peptides were synthesized by Thermo Fisher Scientific (listed in Table 1). One μg of each peptide was coated onto a well of maleimide activated plates (Thermo Fisher Scientific) and then plates were blocked by adding 200 μl of 1% BSA in TBS (pH 7.4) for 1 hour at room temperature. Purified 6xHis-GST-ZO-1 PDZ1 (0.427 pg/ml to 4.27 μg/ml) in 1% BSA in TBS was added to the plates and then plates were incubated for 1 hour at room temperature. Then, plates were washed by 0.5% tween-20 containing TBS (T-TBS) three times and were incubated with HRP-conjugated goat anti-GST (Thermo Fisher Scientific, PA5-141140, 1:5,000). Then, plates were washed by T-TBS five times and were treated with TMB substrate (Thermo Fisher Scientific). The affinity (Kd) was determined using a one-site saturation binding model in Graph Pad Prism 9.

### Western blotting

Cells were lysed by 1% Triton-X in PBS with phosphatase and protease inhibitors (Millipore-Sigma), incubated for 30 minutes on ice and centrifuged at 12,000 g at 4°C for 20 minutes to collect the supernatant (Triton-X soluble fraction). The pellet was washed in 1% Triton-X in PBS with centrifugation and resuspended by 20 μl of 1% Triton-X in PBS. Then, insoluble pellet was lysed by Sample buffer (Final SDS concentration is 2%) with boiling at 95°C for 10 minutes (Triton-X insoluble fraction). Gels were transferred onto methanol-activated polyvinylidene difluoride (PVDF) membranes (Immobilon-P Transfer Membrane, Merck Millipore) via semi-dry transfer. Transferred membranes were blocked under slight agitation in T-TBS with 5% w/v Marvel non-fat dry milk for 1 hour at room temperature. Blocked membranes were treated with primary antibody overnight at 4°C [CLDN-25 (Thermo Fisher Scientific, PA5-91879, 1:2,000), and β-actin (Abcam, ab8227, 1:4,000))]. Membranes were washed three times for 5 minutes in T-TBS and incubated with horse radish peroxidase-conjugated goat anti-rabbit secondary antibody (Sigma-Aldrich) diluted 1:5,000 in T-TBS for 2 hours at room temperature. Secondary antibody was removed and membranes were washed four times for 5 minutes in T-TBS. Membranes were treated with WesternBright ECL Luminol/enhancer solution and Peroxide Chemiluminescent solution (Advansta) for 2 minutes and then chemiluminescence was detected using The LiCor C-Digit Blot Scanner.

### Measurement of the barrier function of CLDN25

To prepare monolayers of CLDN-25 expressing MDCKII cells, MDCKII/*Cldn25* and b.End3/*Cldn25* KO cells, 0.8 × 10^5^ of a series of transfectants were seeded onto cell culture inserts (polyester membrane, 0.4 μm pore size, 0.3 cm^2^ culture area; Sarstedt) and cultured for more than 5 days (top compartment, 0.3 ml; bottom compartment, 1.2 ml). Some blank inserts (without cells) were also cultured. Medium was exchanged every two days.

Trans-epithelial/endothelial electrical resistance (TEER) and dilution potential was measured by using a Millicell ERS Ohmmeter (Millipore). To measure the permeability of sodium fluorescein (FNa) (376 Da; Wako Pure Chemical) and 4 kDa of FITC-dextran (FD4) (Sigma-Aldrich) across the monolayers, cell culture inserts were washed by DMEM (without FBS) and were transferred to 24-well plates (Sarstedt) containing 1.0 ml of DMEM (without FBS). The DMEM in the top chamber was then changed to 0.2 ml of 10 μg/ml FNa or 1 mg/ml of FD4 in DMEM (without FBS), and the culture inserts were incubated for 60 minutes at 37°C. Samples were collected from the bottom compartment, and the concentration of the tracer was measured using FLUOstar OPTIMA plate reader (BMG Labtech). The equations to calculate TEER, apparent permeability of solutes, dilution potential and absolute ion permeability were described previously [30].

### Statistics

For statistical analysis, one-way ANOVA or unpaired t-test was applied using Graph Pad Prism 9. If not stated otherwise, data are given as mean ± SD (^*^P < 0.05; ^**^P < 0.01; ^***^P < 0.001, ^****^P < 0.0001).

## Supplementary Material

Fig_S1_ddae038

Fig_S2_ddae038

S_ddae038_Table_1_ddae038

Supplementary_Legends_ddae038
