# Correction to: A loss of function mutation in *CLDN25* causing Pelizaeus-Merzbacher-like leukodystrophy

**DOI:** 10.1093/hmg/ddaf192

**Published:** 2026-01-05

This is a correction to Yosuke Hashimoto, Claude Besmond, Nathalie Boddaert, Arnold Munnich, Matthew Campbell, A loss of function mutation in *CLDN25* causing Pelizaeus-Merzbacher-like leukodystrophy, *Human Molecular Genetics*, 2024, Volume 33, Issue 12, pp. 1055–1063; https://doi.org/10.1093/hmg/ddae038.

In September 2025, a reader contacted the journal about a potential error in the symbol used for Claudin containing domain 1. The journal contacted all co-authors in line with COPE guidance.

After consideration, the HUGO Gene Nomenclature Committee and Human Genome Variation Society have designated this gene using *CLDND1*. While it may be commonly known as *CLDN25*, according to journal policy, the HUGO Gene Nomenclature should be followed. As such, the following corrections should be made:

1. *CLDN25* should be corrected to CLDND1 in the title.

2. *CLDN25* should be corrected to CLDND1 throughout the article and Supplementary Materials.

3. The abstract is corrected to read: Claudin containing domain 1 (CLDND1), more commonly known as Claudin-25 (CLDN-25), is an uncharacterized claudin family member. It has less conserved amino acid sequences when compared to other claudins. It also has a very broad tissue expression profile and there is currently a lack of functional information from murine knockout models. Here, we report a *de novo* missense heterozygous variant in CLDND1 (*CLDN25*) (c. 745G>C, p. A249P) found in a patient diagnosed with Pelizaeus-Merzbacher-like leukodystrophy and presenting with symptoms such as delayed motor development, several episodes of tonic absent seizures and generalized dystonia.

This correction of the gene symbol does not alter the conclusions reached in the article and the authors agree with the correction.

These details have been corrected only in this correction notice to preserve the published version of record.

