# Supplementary figures and images for: A loss of function mutation in CLDN25 causing Pelizaeus-Merzbacher-like leukodystrophy
Source: Hum Mol Genet. 2024 Mar 17;33(12):1055–63. doi: 10.1093/hmg/ddae038 (PMC11153337; doi:10.1093/hmg/ddae038)

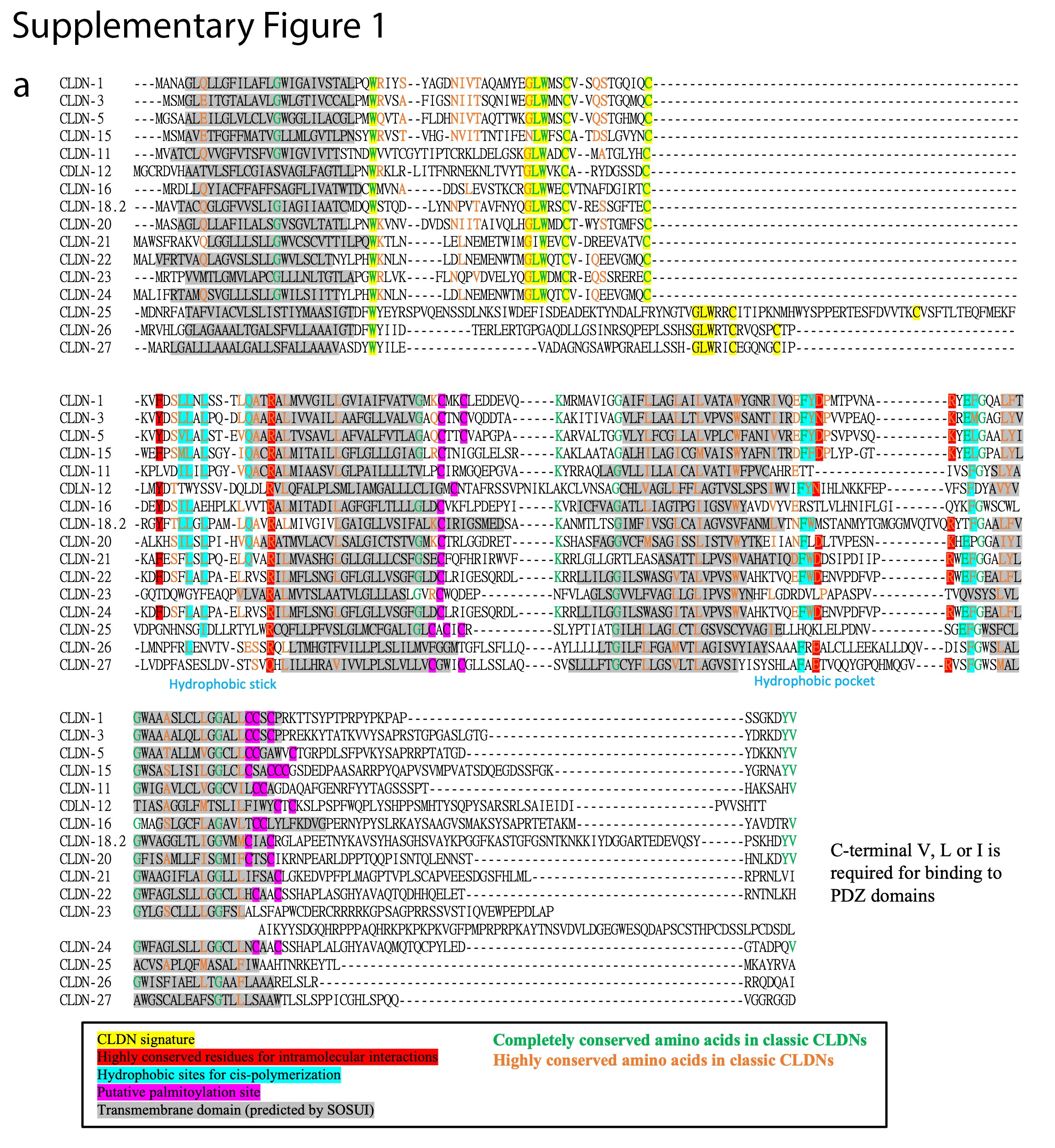

Supplement: Fig_S1_ddae038 [file fig_s1_ddae038.jpeg]

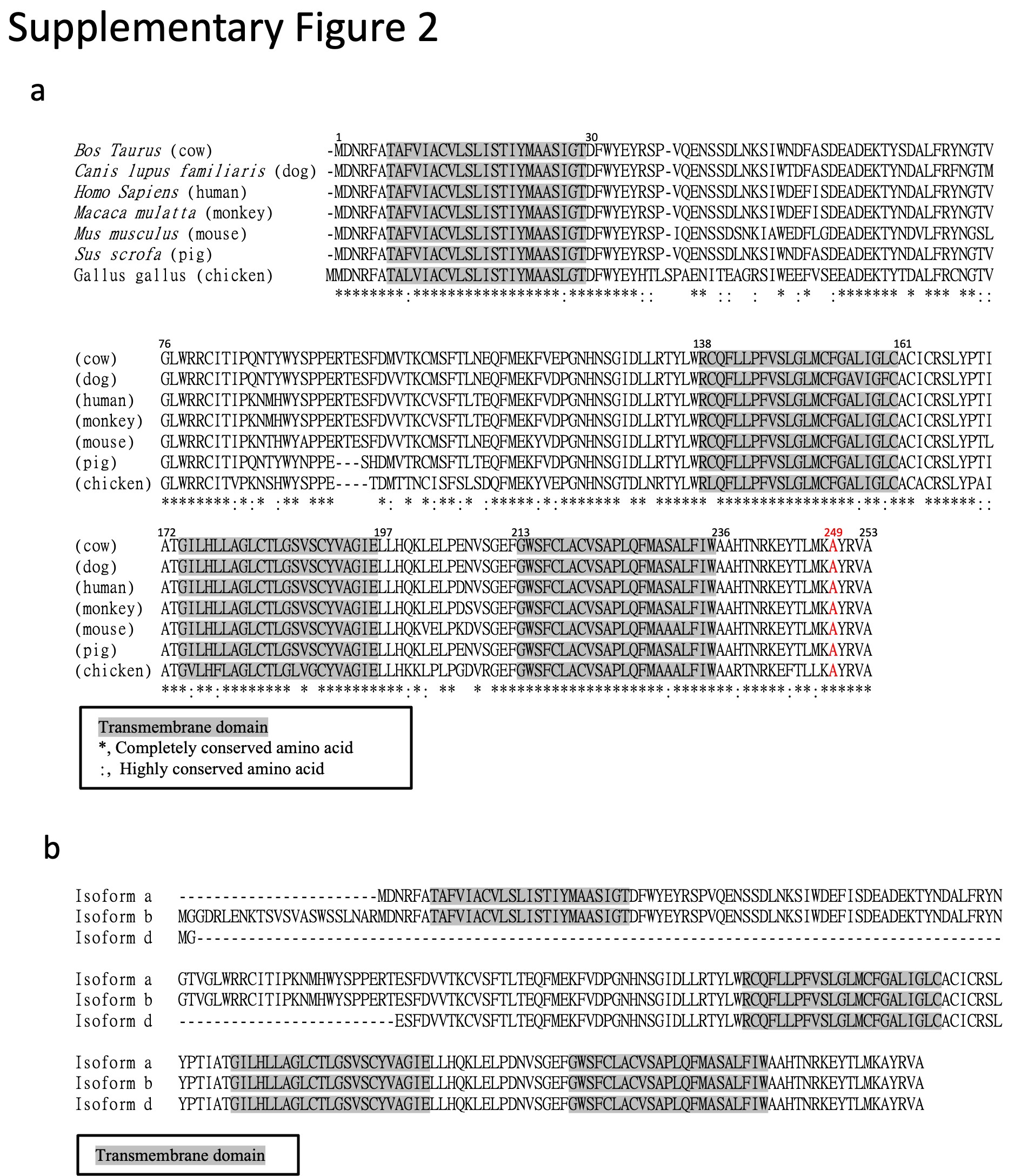

Supplement: Fig_S2_ddae038 [file fig_s2_ddae038.jpeg]
