# Supplementary material for: A loss of function mutation in CLDN25 causing Pelizaeus-Merzbacher-like leukodystrophy
Source: Hum Mol Genet. 2024 Mar 17;33(12):1055–63. doi: 10.1093/hmg/ddae038 (PMC11153337; doi:10.1093/hmg/ddae038)
Supplement: Supplementary_Legends_ddae038 [file supplementary_legends_ddae038.docx]

**Supplementary Figure 1: The sequence alignment of CLDN-25 with the other non-classic CLDNs**

**a)** The extracellular domains of CLDN-5 and -25 are shown by POTTER (http://wlab.ethz.ch/protter/start/). **b)** The sequence alignment of four representative classic human CLDNs (CLDN-1, -3, -5 and -15) and all non-classic CLDNs. The transmembrane domains are predicted by SOSUI system (https://harrier.nagahama-i-bio.ac.jp/sosui/mobile/). Some important amino acids are highlighted.

**Supplementary Figure 2: The supplementary information of amino acid sequence of CLDN-25**

**a)** The sequence alignment of CLDN-25 in different animals. **b)** The sequence alignment of isoforms of human CLDN-25. The transmembrane domains are predicted by SOSUI system.
